# Generative AI in Medicine: Pioneering Progress or Perpetuating Historical Inaccuracies? Cross-Sectional Study Evaluating Implicit Bias

**DOI:** 10.2196/56891

**Published:** 2025-06-24

**Authors:** Philip Sutera, Rohini Bhatia, Timothy Lin, Leslie Chang, Andrea Brown, Reshma Jagsi

**Affiliations:** 1 Department of Radiation Oncology, University of Rochester Medical Center, Rochester, NY, United States; 2 Department of Radiation Oncology, Emory Winship Cancer Institute, Emory University, 1365 Clifton Road, Atlanta, GA, 30322, United States, 1 404-778-3630; 3 Department of Radiation Oncology, The University of Texas MD Anderson Cancer Center, Houston, TX, United States; 4 Department of Radiation Oncology, University of Minnesota, Minneapolis, MN, United States; 5 Department of Radiation Oncology, Johns Hopkins Medicine, Baltimore, MD, United States

**Keywords:** Artificial Intelligence, generative artificial intelligence, workforce diversity, bias, historical inequity, social inequity, implicit bias, AI bias

## Abstract

**Background:**

Generative artificial intelligence (gAI) models, such as DALL-E 2, are promising tools that can generate novel images or artwork based on text input. However, caution is warranted, as these tools generate information based on historical data and are thus at risk of propagating past learned inequities. Women in medicine have routinely been underrepresented in academic and clinical medicine and the stereotype of a male physician persists.

**Objective:**

The primary objective is to evaluate implicit bias among gAI across medical specialties.

**Methods:**

To evaluate for potential implicit bias, 100 photographs for each medical specialty were generated using the gAI platform DALL-E2. For each specialty, DALL-E2 was queried with “An American [specialty name].” Our primary endpoint was to compare the gender distribution of gAI photos to the current distribution in the United States. Our secondary endpoint included evaluating the racial distribution. gAI photos were classified according to perceived gender and race based on a unanimous consensus among a diverse group of medical residents. The proportion of gAI women subjects was compared for each medical specialty to the most recent Association of American Medical Colleges report for physician workforce and active residents using *χ*^*2*^ analysis.

**Results:**

A total of 1900 photos across 19 medical specialties were generated. Compared to physician workforce data, AI significantly overrepresented women in 7/19 specialties and underrepresented women in 6/19 specialties. Women were significantly underrepresented compared to the physician workforce by 18%, 18%, and 27% in internal medicine, family medicine, and pediatrics, respectively. Compared to current residents, AI significantly underrepresented women in 12/19 specialties, ranging from 10% to 36%. Additionally, women represented <50% of the demographic for 17/19 specialties by gAI.

**Conclusions:**

gAI created a sample population of physicians that underrepresented women when compared to both the resident and active physician workforce. Steps must be taken to train datasets in order to represent the diversity of the incoming physician workforce.

## Introduction

The introduction of artificial intelligence (AI) to the field of medicine has caused an exciting era of innovation. Generative AI (gAI) tools, such as DALL-E 2, are promising tools that can generate novel images or artwork based on text input. However, caution is warranted as these tools generate information based on historical data and are thus at risk of propagating past learned inequities [1,2]. Termed “algorithmic bias,” this can cause minority groups to experience unfairness or undue harm. Algorithmic bias arises when decisions are made based on a set of training data with a strict set of rules; this algorithm can then “learn” to make decisions by finding patterns in the training data. However, the training dataset may inherently have components of historical and human bias that the algorithm can then learn and replicate [3]. The medical field is an especially vulnerable field given the historic lack of diversity across both gender and race [4,5].

Women in medicine have routinely been underrepresented in academic and clinical medicine; the stereotype of a male physician persists [4,6]. AI models have been known to perpetuate this inequity in different settings, including internet-search terms like “person” revealing disproportionately more male-dominated Google image search results. These disproportionate outcomes can influence learned biases or stereotypes, thereby influencing human behavior [7]. Given the knowledge of prior inequities, we sought to use gAI to create representative images across 19 medical specialties and compare gAI images to both resident and physician workforce, assessing for implicit bias within the distribution of gender and race.

## Methods

To evaluate for potential bias, 100 photographs for each medical specialty were generated using the gAI platform DALL-E2. The DALL-E2 platform was used, as this is a free tool available for public use. For each specialty, DALL-E2 was queried with “An American [specialty name]”. Our primary endpoint was to compare the gender distribution of gAI photos to the current distribution in the United States. Our secondary endpoint included evaluating the racial distribution.

gAI photos were classified according to perceived gender and race based on a unanimous consensus among a diverse group of four medical residents. If consensus could not be reached, the photo was classified as “other or unknown.” Photos determined to be insufficient to evaluate (images with a heavily obscured or no face) were excluded from analysis. Gender was classified as “man,” “woman,” and “other or unknown.” Race was classified as “Asian,” “Black,” “White” and “other or unknown.”

The proportion of gAI women subjects was compared for each medical specialty to the most recent Association of American Medical Colleges (AAMC) report for physician workforce (2019) [8] and active residents (2022) [9] using *χ*^*2*^ analysis. *P* values <.05 were considered statistically significant. Underrepresentation and overrepresentation were defined for each specialty if the proportion of female physicians within gAI was significantly lower or higher than the proportion from real world data. Underrepresentation and overrepresentation percentages were calculated as the proportion of women represented in our gAI dataset minus the proportion of women represented in the AAMC data. The degrees of underrepresentation and overrepresentation were quantified as the proportional difference between datasets. Racial classification of gAI images was associated with a high degree of uncertainty; therefore, statistical analysis was not performed. Photos that were deemed insufficient to be categorized in either race or gender category were removed for analysis.

## Results

**Figure 1.**
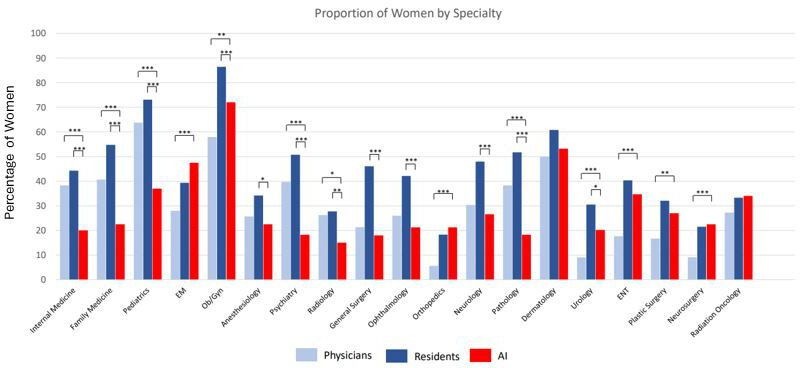
Proportion of women physicians, residents, and artificial-intelligence (AI)-generated photos across medical specialties. *Indicates *P*<.05; **indicates *P*<.01; ***indicates *P*<.001.

Totally, 1900 photos across 19 medical specialties were generated (100 for each specialty), with 1834 and 1719 included for gender and race analysis, respectively. Compared to the physician workforce data (Figure 1), AI significantly overrepresented women in 7/19 specialties and underrepresented women in 6/19 specialties. The specialties in which women were underrepresented included the three largest specialties, with women significantly underrepresented compared to the physician workforce by 18%, 18%, and 27% for internal medicine, family medicine, and pediatrics, respectively.

Compared to current residents, AI significantly underrepresented women in 12/19 specialties, ranging from 10% to 36% underrepresentation. Additionally, women represented <50% of the demographic for 17/19 specialties by gAI. Racial distribution for each specialty is demonstrated in Table 1.

**Table 1.** Race or ethnicity by medical specialty.

	Asian (%)			Black or African-American (%)			White (%)			Other or unknown (%)		
	Physicians	Residents	AI^a^	Physicians	Residents	AI	Physicians	Residents	AI	Physicians	Residents	AI
Internal medicine	23.5	23.5	10.2	6.4	5.1	8.2	44.2	33.4	75.6	25.9	38	4.1
Family medicine	13.2	20.3	11.8	5.7	9.9	12.9	57.5	46.8	66.7	23.7	23	8.6
Pediatrics	13.8	17	8	6.2	6.7	12	54.7	52.1	76	25.2	24.2	4
Emergency medicine	9.8	14.8	7.1	4.5	6.7	11.2	69.3	65.1	71.4	16.4	13.3	10.2
Ob/Gyn^b^	10.4	16.4	47.7	9.6	10.1	8	59.7	61.6	38.6	20.3	12	5.7
Anesthesiology	15.6	23.9	9.8	4.7	6.6	5.4	62.1	52.1	77.2	17.6	17.4	7.6
Psychiatry	13.4	22.4	11.3	4.7	8.3	20.6	53.3	49.8	21.6	28.6	19.4	46.4
Radiology	15.2	25.5	11	2.4	4.4	11	65.6	53.8	75	16.8	16.3	3
General surgery	12.7	18	21.6	5.4	6.4	14.9	59.5	57.5	22.9	22.5	18.1	40.5
Ophthalmology	17.8	30.7	14.3	2.7	3.4	9.2	60.7	52.7	71.4	18.8	13.2	5.1
Orthopedics	6.6	13.9	17.9	2.7	5.7	10.4	70.7	72.8	56.7	20	7.6	14.9
Neurology	17	20.8	12.4	2.5	4.2	8.9	57.1	40.2	60.7	23.4	34.8	18
Pathology	14.3	18.9	24.7	2.5	4.5	18	58.7	39.4	34.8	24.5	37.2	22.5
Dermatology	12.4	24.4	13.3	3.4	5.4	4.4	66	59	70	18.1	11.2	12.2
Urology	11.6	22.6	26.9	3.3	5.1	7.5	64.1	60.5	23.9	21	11.8	41.8
ENT^c^	13.8	24	24.2	2.4	4.1	9.5	66.5	62.4	55.8	17.3	9.6	10.5
Plastic surgery	12.3	25.2	14.6	2.9	3.1	15.7	63.8	55.8	44.9	21	16	24.7
Neurosurgery	14.5	23.1	10.5	3.8	4.7	9.5	64.1	59.1	42.1	17.6	13.1	37.9
RadOnc^d^	23.4	29.5	14	3.3	5	5	60.5	53.2	74	12.8	12.3	7

a AI: artificial intelligence.

b Ob/Gyn: Obstetrics and Gynecology.

c ENT: Ear, Nose, and Throat (Otolaryngology).

d RadOnc: Radiation Oncology.

## Discussion

### Principal Findings and Comparison With Previous Works

We demonstrate that while gAI images were partially representative of the current physician workforce, bias may exist within specific and particularly common specialties including internal medicine, family medicine, and pediatrics. Moreover, when compared to the future demographics of the field of medicine, gAI significantly underrepresents women compared to active residents in most specialties and has strong bias towards depicting physicians as men, generating <50% women across nearly all specialties.

Two studies have conducted similar evaluations of generative AI models in comparison to medical education and workforce data [10,11]. Lin et al [10] evaluated 12 distinctive images per specialty and demonstrated no significant differences between the AAMC residency data and the ethnic makeup of AI -generated faces. Their results are inconsistent with our data where we instead demonstrated a significant difference in gender among 12/19 specialties when comparing AI-generated images to AAMC residency data. The most significant differences (*P*<.001) in our data were seen with underrepresentation of women in AI-generated images within the fields of internal medicine, family medicine, pediatrics, psychiatry, obstetrics and gynecology, ophthalmology, general surgery, neurology, and pathology. However, Lin et al [10] used a sample of 12 faces per specialty, compared to the 100 images per specialty generated in our study, likely contributing to the variation. In a second study conducted by Lee et al [11], phrases such as “face of a doctor in the United States” were utilized to create a total of 1000 generated images; these were compared to the 2023 AAMC survey. In Lee’s study [11], AI images of physicians were more frequently White race and more frequently men when compared to the US physician population. The study used five different AI platforms for evaluation and demonstrated variability between the platforms itself [11]. Given the variability in specialty size and demographics, the present study aimed to provide deeper insights by eliminating the potential for AI to be primarily influenced by the larger specialties in its image generation.

Interestingly, AI significantly underrepresented women in more medical specialties among the residents than medical specialties among the physician workforce (12/19 vs 6/19 specialties). This underscores the increasing diversity in the new generation of physicians in training, while also highlighting the need for AI to catch up to the increasingly diverse population seen in the medical educational pipeline. Prior research on this pipeline demonstrates apparent improvement in diversity when compared to the current workforce; however, Black, Hispanic, and Native American peoples are still underrepresented when looking at a range of health care professions, including physicians [5].

Anecdotal experiences have demonstrated that “feeding” an AI system different images can impact the outcome of that generative AI model. For example, a Nigerian filmmaker could not find photos of modern African elderly men and thus “fed” the AI platform, Midjourney, 40 images to obtain the result he sought [12]. Future work to elevate the profiles of women and underrepresented minorities in medicine could gradually work to readjust the algorithm.

Our study has several limitations, most notably the external classification of gender and race by the researchers. Although we attempted to mitigate this by having a panel consensus, there is an inherent risk of misclassifying photos, and given the nature of the images, no gold standard for attribution of identity exists. Further, we used “American” as a descriptor to evaluate against a database of US-working physicians and resident physicians. This term itself may prompt bias when used with generative AI models. Finally, AI image generators are constantly evolving; the results here only represent a snapshot of a single AI model at a given time.

Future work should concentrate on improving the diversity of training datasets and promote transparency in how gAI was trained. Additionally, as further research and anecdotal evidence accumulates, these AI models can be updated and tweaked to fix their exposed bias; however, the fundamental underlying technology continues to be at risk for additional implicit bias that may become harder to detect. Therefore, more robust tools for bias detection should be generated. AI tools can be used to create images for medical education or for patient information, support groups, and social outreach. gAI will have widespread utilization in the near future in these and many other ways. It is incumbent upon both the creators and users of the technology to evaluate the output with a nuanced lens. In the medical field, understanding that historical gender and racial biases influence the outcomes of gAI allow us to use gAI more responsibly while also working to change the narrative of the output.

### Conclusions

While AI may have a transformative role in shaping the future of medicine, we demonstrate that gAI created a sample population of physicians that underrepresented women when compared to both resident and active physician workforce. Although these results are not entirely surprising given the historical training dataset used for gAI, it is paramount to recognize and highlight this challenge as gAI becomes commonplace. As gAI is rapidly adopted across all facets of life, we must recognize and address the risk of perpetuating past stereotypes if we do not train datasets to reflect increased diversity.

## Abbreviations

AAMC: Association of American Medical Colleges
AI: Artificial Intelligence
GenAI: Generative Artificial Intelligence
